# Fingolimod as a Neuroprotective Agent in Ischemic Stroke: A Review of Preclinical and Clinical Evidence

**DOI:** 10.3390/jcm14196797

**Published:** 2025-09-25

**Authors:** Alin Ciubotaru, Roxana Covali, Cristina Grosu, Daniel Alexa, Esthir Flavia Pilă, Andrei Ionuț Cucu, Amelian Madalin Bobu, Gabriela Dumachita Sargu, Laura Riscanu, Mihaela Camelia Tirnovanu, Cristina Adam, Radu Popa, Cristiana Filip, Emilian Bogdan Ignat

**Affiliations:** 1 Department of Neurology, Grigore T. Popa University of Medicine and Pharmacy, 700115 Iasi, Romania; alinciubotaru94@yahoo.com (A.C.);; 2 Department of Radiology, Biomedical Engineering Faculty, Grigore T. Popa University of Medicine and Pharmacy, 700115 Iasi, Romania; 3 Department of the Faculty of Medicine, Grigore T. Popa University of Medicine and Pharmacy, 700115 Iasi, Romania; 4 Faculty of Medicine and Biological Sciences, University Stefan cel Mare of Suceava, 720229 Suceava, Romania; andrei.cucu@usm.ro; 5 Department of Cardiology, Saint Spiridon Hospital, 700111 Iasi, Romania; 6 Department of Obstetrics and Gynecology, Elena Doamna Obstetrics and Gynecology University Hospital, Grigore T. Popa University of Medicine and Pharmacy, 700115 Iasi, Romania; sargu_gabriela@yahoo.com; 7 Department of Morphofunctional Sciences, Grigore T. Popa University of Medicine and Pharmacy, 700115 Iasi, Romania; 8 Mother and Child Department, Grigore T. Popa University of Medicine and Pharmacy, 700115 Iasi, Romania; mihaela.tirnovanu@umfiasi.ro; 9 Department of Medical Specialties I, Grigore T. Popa University of Medicine and Pharmacy, University Street No. 16, 700115 Iasi, Romania; 10 Clinical Rehabilitation Hospital, Cardiovascular Rehabilitation Clinic, Pantelimon Halipa Street No. 14, 700661 Iasi, Romania; 11 Department of Vascular Surgery, Grigore T. Popa University of Medicine and Pharmacy, 700115 Iasi, Romania; 12 Department of Biochemistry, Grigore T. Popa University of Medicine and Pharmacy, 700115 Iasi, Romania; cristiana.filip@umfiasi.ro

**Keywords:** fingolimod, ischemic stroke, neuroprotection, sphingosine-1-phosphate receptors, neuroinflammation, blood-brain, barrier

## Abstract

Ischemic stroke remains a leading cause of mortality and disability worldwide, with current therapies such as intravenous thrombolysis and mechanical thrombectomy benefiting only a limited proportion of patients. Neuroinflammation is a key contributor to secondary brain injury, creating a strong rationale for adjunctive therapies targeting immune modulation. Fingolimod, a sphingosine-1-phosphate receptor (S1PR) modulator originally approved for multiple sclerosis, has shown promising effects in both preclinical and early clinical studies of acute ischemic stroke. Methods: We conducted a structured narrative review of preclinical and clinical studies published between 2015 and 2024, using PubMed, Scopus, and Web of Science databases. Inclusion criteria were original studies evaluating fingolimod in ischemic stroke models or human patients, either as monotherapy or in combination with reperfusion therapies. Exclusion criteria included conference abstracts without peer review, studies lacking mechanistic insight, and non-English publications. Results: Preclinical evidence demonstrates that fingolimod reduces infarct size, preserves blood–brain barrier integrity, and modulates neuroinflammation through multiple mechanisms, including T cell sequestration, microglial polarization, and mitochondrial protection. Clinical trials, though limited in size, suggest improved short- and long-term outcomes when fingolimod is used in combination with intravenous thrombolysis or endovascular therapy, with a manageable safety profile. Novel nanotechnology-based delivery systems further enhance central nervous system (CNS) targeting and reduce systemic side effects. Conclusions: Fingolimod represents a promising multi-targeted adjunctive strategy for ischemic stroke, acting at the intersection of immune modulation, vascular protection, and neuroprotection. While current findings are encouraging, larger randomized controlled trials and biomarker-driven patient selection are needed to validate its clinical utility. This review highlights the translational potential of fingolimod and outlines key directions for future research.

## 1. Introduction

Stroke remains a leading cause of mortality and disability worldwide, ranking as the third leading cause of death in 2021. According to the Global Burden of Disease Study 2021, ischemic strokes accounted for approximately 65.3% of all stroke cases globally [1].

The pathophysiology of ischemic stroke is multifaceted, initiated by excitotoxicity, oxidative stress, energy failure, and programmed cell death [2]. Neuroinflammation plays a central role, amplifying tissue injury via swift activation of resident microglia followed by infiltration of peripheral immune cells. This acute inflammatory process directly disrupts the blood–brain barrier (BBB) by upregulating matrix metalloproteinases (MMP-9), adhesion molecules (ICAM-1), VEGF, and oxidative stress, leading to loss of tight junction integrity, vasogenic edema, and secondary neuronal damage [3].

Current therapies for ischemic stroke, such as intravenous thrombolysis with recombinant tissue plasminogen activator (rt-PA) and mechanical thrombectomy, are highly time-dependent and benefit only a limited subset of patients. For instance, thrombolysis must be administered within 4.5 h of symptom onset and provides functional recovery in only a modest proportion of cases [4]. While thrombectomy improves outcomes in large-vessel occlusions, less than 15–20% of stroke patients are eligible, with access rates as low as 2.5% in some European countries [5,6]. Despite extensive preclinical efforts, no neuroprotective agents have yet received regulatory approval, largely due to translational gaps and clinical trial failures [7].

Given the narrow time window and risks of reperfusion therapies, there is an urgent need for adjunctive neuroprotective agents that can reduce ischemic injury, preserve neuronal function, and extend eligibility for reperfusion treatments [8]. Moreover, in accord with systematic reviews, most single-target neuroprotective agents failed in clinical trials due to issues such as poor BBB penetration, heterogeneous stroke pathophysiology, and delayed drug administration [7,9]. To overcome these issues, novel delivery systems like platelet membrane-coated fingolimod nanobubbles (PFNBs) and neutrophil-mimicking nanoreactors have been designed to improve CNS targeting and exploit post-stroke inflammation. Notably, fingolimod, an S1P receptor modulator, has shown encouraging results in early trials when combined with reperfusion, improving The National Institutes of Health Stroke Scale (NIHSS) scores and 90-day outcomes, while maintaining safety [10].

Despite being a leading cause of global mortality and disability, the therapeutic arsenal for acute ischemic stroke (AIS) remains limited to reperfusion strategies, which are restricted by narrow time windows and eligibility criteria [4,5,6]. A central reason for the repeated failure of neuroprotective agents in clinical translation is the multifaceted nature of ischemic brain injury, which evolves over time and involves interconnected pathological cascades [7,9]. Beyond the initial excitotoxic insult, the secondary phase of injury is dominated by two critically intertwined processes: maladaptive neuroinflammation and blood–brain barrier (BBB) disruption. These processes amplify each other, leading to progressive tissue damage, cerebral edema, hemorrhagic transformation, and ultimately, worse neurological outcomes [3]. Therefore, an effective neuroprotective strategy must conceptually address this neurovascular-immune axis.

Fingolimod (FTY720), a sphingosine-1-phosphate (S1P) receptor modulator approved for multiple sclerosis, has emerged as a promising candidate that directly targets this conceptual framework. Its known mechanisms of action—immunomodulation and BBB stabilization—align precisely with the key pathologies driving secondary injury in stroke. This review synthesizes the preclinical and clinical evidence for fingolimod, evaluating its potential as a multi-targeted therapeutic agent designed to interrupt the thrombo-inflammatory cascade in AIS.

### 1.1. Elements of Novelty of This Review

Recent and Comprehensive Scope

The review compiles the latest (2015–2024) preclinical evidence on Fingolimod’s role in ischemic stroke, including models with comorbidities like aging and diabetes.

Multidimensional Mechanistic Insight

It synthesizes Fingolimod’s diverse mechanisms: modulation of inflammation (High Mobility Group Box 1HMGB1/TLR4 Toll-Like Receptor 4/NF-κB Nuclear Factor Kappa-Light-Chain-Enhancer of Activated B Cells), Treg activation, microglial polarization, BBB preservation, and autophagy suppression.

Integration of Imaging and Molecular Data

A novel aspect is the inclusion of imaging biomarkers (e.g., Ultrasmall Superparamagnetic Iron Oxide—Susceptibility Weighted Imaging USPIO-SWI) alongside molecular data for a comprehensive evaluation.

Model-Based Comparative Analysis

The review compares Fingolimod’s efficacy across various stroke models (transient Middle Cerebral Artery Occlusion (tMCAO), permanent Middle Cerebral Artery Occlusion (pMCAO), Prothrombin Time PT, etc.) and contexts, providing valuable translational insights.

Emphasis on Subacute and Functional Outcomes

Unlike previous work, this review highlights studies assessing neurological recovery beyond the acute phase.

Critical Review of Safety and Limitations

It underscores translational challenges, including administration routes, dosing, immunosuppression risk, and model-specific discrepancies.

Therapeutic Outlook

The review supports future clinical strategies, such as combining Fingolimod with reperfusion therapies, referencing emerging trials (e.g., Combinating Fingolimod With Alteplase Bridging With Mechanical Thrombectomy in Acute Ischemic Stroke (FAMTAIS)).

### 1.2. Motivation of the Study

The restoration of cerebral blood flow (CBF) is essential in the management of acute ischemic stroke. However, when recanalization is delayed beyond the critical therapeutic window, the abrupt influx of oxygen-rich blood into metabolically compromised tissue may overwhelm endogenous antioxidant defenses and further damage the already impaired vascular endothelium. This phenomenon, known as reperfusion injury, involves oxidative stress, disruption of the blood–brain barrier (BBB), and neuroinflammation. Inflammatory responses amplify BBB breakdown and promote immune cell infiltration, exacerbating neuronal damage.

Given the limitations of current treatments in addressing post-ischemic inflammatory cascades, our review aims to synthesize the existing preclinical and clinical evidence on fingolimod, a sphingosine-1-phosphate receptor (S1PR) modulator, as a neuroprotective agent in ischemic stroke. Recent studies suggest that combining anti-inflammatory agents with rt-PA, the standard thrombolytic therapy, can improve outcomes [11,12,13,14]. We were motivated by the drug’s multifaceted mechanisms of action—particularly its ability to modulate immune responses, stabilize the blood–brain barrier, and reduce secondary injury following stroke. Its immunomodulatory effects that help preserve BBB integrity could reduce infarct volume, hemorrhagic risk, and neurological deficits when used alongside rt-PA [15,16]. These findings support the development of multi-targeted therapeutic strategies that address both reperfusion and inflammation.

### 1.3. Objectives of Study

The main objective of this review is to critically evaluate how fingolimod has been studied across various experimental models and clinical settings, both as a monotherapy and in combination with rt-PA, to assess its potential for translation into broader clinical use. Furthermore, we explore the limitations and challenges associated with fingolimod, including adverse effects and inconsistent outcomes and present emerging strategies such as nanotechnology-based delivery systems that may overcome these barriers. By identifying both the therapeutic potential and the current gaps in knowledge, this review contributes to the ongoing effort to develop effective adjunctive treatments that target inflammation in acute ischemic stroke.

In sum, ischemic stroke remains a global health priority, with high mortality and disability rates and limited access to effective reperfusion therapies. The multifactorial nature of stroke injury, particularly the central role of neuroinflammation and blood–brain barrier disruption, underscores the urgent need for novel neuroprotective strategies. Fingolimod emerges as a promising candidate due to its ability to modulate immune responses, stabilize vascular integrity, and reduce secondary injury, especially when used alongside reperfusion therapies. The novelty of this review lies in synthesizing recent mechanistic insights, imaging biomarkers, and translational studies, while also addressing safety challenges and delivery innovations. Together, these aspects highlight fingolimod’s potential to bridge the translational gap and support the development of multi-targeted approaches in acute ischemic stroke.

## 2. Materials and Methods

This narrative review was conducted to summarize and critically evaluate the existing body of literature on the neuroprotective potential of fingolimod in ischemic stroke. Given the rapid development of this field and the aim to provide a contemporary overview, we focused on synthesizing evidence from the last decade.

### 2.1. Search Strategy and Data Sources

A structured literature search was performed to identify relevant preclinical and clinical studies. Primary electronic databases consulted included PubMed/MEDLINE and ScienceDirect. To ensure coverage of key outcomes research in the field, we also searched Medicina (Basel).

The search strategy was designed to capture the core concepts of the review. Search terms included combinations of: “fingolimod” OR “FTY720” AND “ischemic stroke” OR “cerebral ischemia” AND “neuroprotection” OR “neuroinflammation” OR “blood-brain barrier”. Boolean operators (AND, OR) were used to refine the results.

Furthermore, to mitigate the risk of omission and identify seminal works, we performed a secondary, iterative search strategy. This included:

Manual screening of reference lists from all included full-text articles and key previous reviews on sphingosine-1-phosphate modulators in stroke.

Citation tracking (forward and backward searching) of pivotal primary studies and clinical trials to identify additional relevant literature. To ensure a comprehensive retrieval of records, a secondary, iterative search strategy was employed. Manual screening of reference lists from all included full-text articles and key previous reviews on the topic. Citation tracking (both backward and forward searching) of pivotal primary studies and clinical trials to identify additional relevant literature that may not have been captured by the initial database search.

### 2.2. Inclusion and Exclusion Criteria

Inclusion criteria were:

Studies evaluating fingolimod as a neuroprotective agent in models of ischemic stroke (both in vivo and in vitro) or in human subjects.

All study types, including original research articles, pilot clinical trials, randomized controlled trials (RCTs), and non-randomized studies.

Studies reporting outcomes such as infarct volume, neurological scores (e.g., NIHSS, mRS), inflammatory markers, or BBB integrity.

Research on novel fingolimod delivery systems (e.g., nanoparticle-based formulations).

Publications in English with full-text availability.

Exclusion criteria were:

Studies unrelated to stroke or neuroprotection.

Non-English publications.

Abstracts, editorials, or conference proceedings without accompanying full-text, original data.

Articles focused exclusively on hemorrhagic stroke, multiple sclerosis, or other non-neuroprotective indications.

### 2.3. Study Selection and Data Extraction

All identified titles and abstracts underwent an initial screening for relevance. Articles deemed potentially eligible were retrieved in full text and independently assessed by two reviewers for final inclusion based on the criteria above. Any disagreements were resolved through discussion until a consensus was reached.

Data extracted from each article included: first author, year of publication, study type (e.g., preclinical animal study, pilot RCT, RCT), model or population characteristics, dosage and administration scheme of fingolimod, primary outcomes, and main conclusions.

As this is a narrative review, no formal systematic review protocols (e.g., PRISMA) or quality assessment tools (e.g., SYRCLE for animal studies, Cochrane RoB 2 for trials) were applied. However, the scientific validity of included studies was considered based on methodological rigor, sample size, and clarity of reported findings.

## 3. Epidemiology and Pathophysiology in AIS

Cerebral ischemia is the second leading cause of death globally and remains a major contributor to both high mortality rates and long-term disability [17]. In 2021, ischemic stroke resulted in an age-standardized mortality rate (ASDR) of approximately 44.2 per 100,000 individuals, according to the Global Burden of Disease (GBD) data [18].

In cerebral ischemia, the interruption of oxygen and glucose supply leads to cellular injury and metabolic failure. If blood flow is later restored, especially after a prolonged ischemic episode, a paradoxical phenomenon known as reperfusion injury may occur, in which the returning oxygen-rich blood exacerbates brain damage rather than alleviating it. A major contributor to this secondary injury is neuroinflammation, triggered by multiple mechanisms. Reperfusion rapidly introduces reactive oxygen species (ROS) into the ischemic tissue, which not only causes oxidative damage but also activates immune signaling [15]. Necrotic and damaged brain cells release intracellular components known as damage-associated molecular patterns (DAMPs), including nucleic acids, which bind to immune receptors like Toll-like receptors (TLRs) [19]. This immune activation stimulates microglial cells, promotes the release of pro-inflammatory cytokines and chemokines, and recruits peripheral immune cells into the brain. These infiltrating leukocytes and platelets can block microvessels, worsen local hypoperfusion, and amplify neuronal injury. Activated immune cells also produce additional ROS and nitric oxide (NO) via inducible nitric oxide synthase (iNOS), compounding the damage. Moreover, neuroinflammation contributes to blood–brain barrier (BBB) breakdown, brain edema, and the risk of hemorrhagic transformation. Thus, while reperfusion is essential for restoring cerebral blood flow (CBF), it can paradoxically initiate a vicious cycle of inflammation and oxidative stress, leading to more extensive tissue damage [15].

## 4. Inflammation: A Common Acute Target in Stroke

According to Kleinig and Vink, inflammation is recognized as a key factor influencing outcomes after acute central nervous system (CNS) injury, as it may significantly contribute to secondary damage [20]. Both in ischemia and after hemorrhage, microglial activation, leukocyte infiltration, the production of pro-inflammatory cytokines (IL-1β, TNF-α) and ROS contribute to the destruction of the blood–brain barrier (BBB) and the increase in cerebral edema [21]. In ischemic stroke the inflammatory response is closely linked to immune system activity [22]. Given the strong interplay between inflammation and immune modulation in this pathology, current treatment approaches prioritize interventions that suppress inflammation, regulate immune cell activity, and disrupt pro-inflammatory signaling pathways to mitigate stroke progression.

Importantly, cerebral ischemia and reperfusion injury highlight the double-edged nature of restoring blood flow: while essential for survival, it triggers oxidative stress, immune activation, and blood–brain barrier disruption that worsen tissue damage. Neuroinflammation is central to this process, with microglial activation, leukocyte infiltration, and cytokine release amplifying injury and contributing to edema and hemorrhagic risk. These insights emphasize that inflammation is not only a consequence but also a driver of poor stroke outcomes, making it a critical therapeutic target for adjunctive treatments.

## 5. Fingolimod Mechanism of Action

Sphingosine 1-phosphate (S1P) is a widely distributed bioactive sphingolipid that exerts a range of essential biological effects, primarily through binding to its five specific G-protein-coupled receptors (S1P1–S1P5). Additionally, it can function as an intracellular signaling molecule [17]. Synthesized by sphingosine kinases (SphK1 and SphK2), S1P is highly concentrated in various tissues, particularly in the brain.

While the therapeutic role of receptor-mediated sphingosine-1-phosphate (S1P) signaling is well established in multiple sclerosis, where FTY720 (fingolimod, Gilenya^™^, Novartis Basel, Switzerland), a non-selective S1P receptor modulator, is widely used, its relevance extends notably to cerebral ischemia. In both preclinical stroke models and clinical studies, fingolimod has demonstrated promising efficacy. Notably, S1P receptors such as S1P1, S1P2, and S1P3 have been implicated as key mediators in the pathogenesis of ischemic brain injury, further underscoring the therapeutic potential of targeting this pathway in stroke. S1P1 is involved in maintaining endothelial barrier integrity and modulating lymphocyte trafficking, how S1P2 contributes to vascular permeability and pro-inflammatory signaling, and how S1P3 is associated with vascular tone regulation and leukocyte infiltration [23].

**Figure 1:**
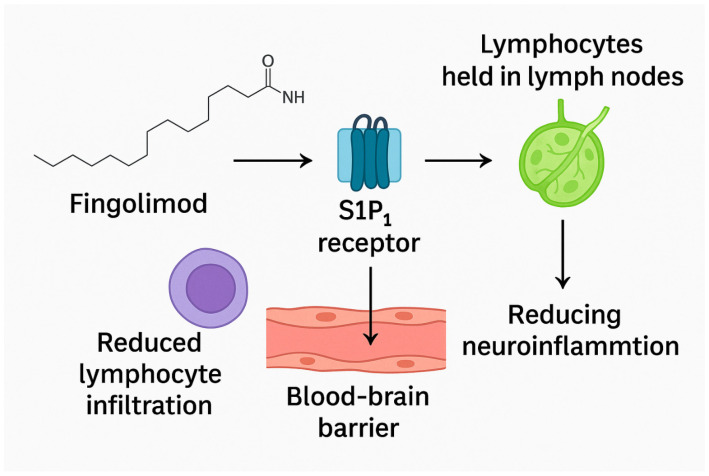
Fingolimod mechanism of action (created in BioRender v2.5. Ciubotaru, A. https://BioRender.com/fi46ba8, accessed on 5 August 2025).

Fingolimod functions as a modulator of sphingosine-1-phosphate receptors (S1PRs), effectively sequestering lymphocytes within lymph nodes and limiting their migration. Once activated through phosphorylation, fingolimod binds to S1PR (sphingosine-1-phosphate receptor) on the surface of CD4+ T cells, effectively causing these receptors to lose their activity. This binding action leads to the sequestration of CD4+ T cells within lymphoid organs, thereby preventing their migration into the brain [24]. By preventing immune cells from gathering in the central nervous system post-ischemia, fingolimod could combat thrombo-inflammation. It also helps mitigate mitochondrial dysfunction, a key contributor to the generation of reactive oxygen species (ROS) [22]. Moreover, when given alongside rtPA within 4.5 h of stroke onset, fingolimod led to a greater reduction in circulating lymphocytes, decreased lesion size, reduced hemorrhagic complications, and improved neurological outcomes compared to treatment with alteplase alone (Figure 1) [25].

Fingolimod (FTY720) is a structural analog of sphingosine that undergoes phosphorylation by sphingosine kinase 2 to form the active metabolite fingolimod-phosphate. This metabolite functions as a high-affinity modulator of sphingosine-1-phosphate receptors (S1PRs), particularly S1P1, S1P3, S1P4, and S1P5. The primary immunological mechanism involves functional antagonism of S1P1 receptors on lymphocytes, resulting in their sequestration within lymphoid tissues and a subsequent reduction in peripheral lymphocyte circulation. This action limits the infiltration of immune cells into the ischemic brain, thereby attenuating post-stroke neuroinflammation.

Beyond its immunomodulatory role, fingolimod exerts direct effects within the central nervous system. Engagement of S1P receptors on endothelial cells enhances blood–brain barrier integrity and reduces vascular permeability, mitigating ischemia-induced edema. In addition, fingolimod has been shown to decrease astrogliosis and microglial activation, modulate cytokine release, and reduce oxidative stress, collectively contributing to neuroprotection. Evidence from preclinical studies also suggests that fingolimod promotes oligodendrocyte survival and remyelination, supporting functional recovery after ischemic injury.

Importantly, fingolimod exerts its neuroprotective potential in ischemic stroke by modulating sphingosine-1-phosphate receptors, reducing harmful immune cell trafficking, and mitigating thrombo-inflammation. By sequestering lymphocytes and limiting their infiltration into the brain, it preserves blood–brain barrier integrity and reduces secondary injury. Beyond immune modulation, fingolimod also impacts mitochondrial function and oxidative stress, further protecting neural tissue. Clinical and preclinical findings suggest that when combined with reperfusion therapies, fingolimod enhances neurological outcomes and reduces complications, highlighting its promise as a multifaceted adjunctive therapy in stroke.

## 6. Preclinical Evidence

We reviewed several high-quality animal studies exploring the mechanisms and efficacy of Fingolimod. A recent preclinical study by Xing et al. (2024) demonstrated that Fingolimod significantly reduced infarct volume, brain edema, and neurological deficits in a rat tMCAO/reperfusion model [26]. The neuroprotective effect was dose-dependent and associated with suppression of pro-inflammatory cytokines (IL-1β, IL-6, TNF-α) and inhibition of the HMGB1/TLR4/NF-κB signaling pathway in the hippocampus. These findings highlight Fingolimod’s potential as an anti-inflammatory therapeutic strategy in acute ischemic stroke. Building on earlier findings that fingolimod increases regulatory T cell frequency after cerebral ischemia [27], the subsequent study further demonstrates that fingolimod also restores Treg suppressive function, suggesting a dual role in quantitative and functional immune modulation post-stroke [28].

A study by Díaz Díaz et al. (2022) suggests that fingolimod’s benefits in stroke are less pronounced than previously reported and may be influenced by the animals’ underlying inflammatory state [29].

In a preclinical embolic stroke model, Fu et al. (2022) demonstrated that the combination of fingolimod and alteplase significantly enhanced cerebral reperfusion, as visualized by USPIO-enhanced SWI MRI [30]. Multiple studies demonstrated fingolimod’s neuroprotective effects in experimental stroke through distinct mechanisms. Li et al. showed anti-inflammatory effects and pro-survival signaling in diabetic tMCAO mice, though without early functional improvement [31]. Wang et al. found that fingolimod preserved BBB integrity, reduced infarct size and mortality, and improved outcomes via S1P_1_/ERK1/2 signaling [32]. Shang et al. reported that fingolimod promoted M2 microglial polarization, enhanced angiogenesis, and supported functional recovery post-stroke [33]. Together, these findings highlight fingolimod’s multifaceted actions on inflammation, microglia, and vascular protection.

Two studies from 2019 provide mechanistic support for later findings on fingolimod’s neuroprotective effects. Salas-Perdomo et al. showed that fingolimod reduces hemorrhagic transformation after stroke, reduces lymphocyte infiltration, and preserves endothelial junctions, though its attenuation effect of hemorrhagic transformation (HT) was platelet-dependent [34]. Ji et al. demonstrated that fingolimod drives microglial polarization toward an M2 phenotype via the nuclear SphK2–S1P axis and epigenetic modulation [35]. Together, these results reinforce subsequent 2020 studies by confirming that fingolimod modulates both immune cell trafficking and microglial function in stroke.

Earlier experimental data from 2017 to 2018 further elucidate fingolimod’s complex and context-dependent actions in ischemic brain injury. Li et al. (2017) showed that fingolimod reduces neuronal autophagy and apoptosis via activation of the mTOR/p70S6K pathway, resulting in smaller infarct volumes and improved functional outcomes [36]. Dong et al. (2018) added mechanistic insight at the astrocytic level, revealing that phosphorylated fingolimod acts through S1PR3 to suppress TLR2/4–NF-κB signaling, thereby attenuating neuroinflammation following oxygen-glucose deprivation [37]. In contrast, Herz et al. (2018) reported that systemic T cell depletion using fingolimod or anti-CD3 antibodies worsened hypoxic–ischemic injury in neonatal mice, suggesting that a complete loss of peripheral T cells may shift the immune balance toward harmful innate immune activation [38].

Research efforts conducted between 2015 and 2016 provided key insights into fingolimod’s protective actions in stroke. Moon et al. (2015) showed that blocking S1P signaling reduces inflammation and infarct size, highlighting its therapeutic potential [39]. Schuhmann et al. (2016) found that fingolimod preserves microvascular patency and improves motor outcomes without affecting glial responses [40]. Similarly, Nazari et al. (2016) reported improved memory and synaptic plasticity after MCAO, indicating benefits beyond structural protection [41]. These findings anticipated later work by emphasizing fingolimod’s vascular, anti-inflammatory, and cognitive effects, Table 1.

Importantly, preclinical studies consistently demonstrate fingolimod’s multifaceted neuroprotective actions in ischemic stroke, spanning immune modulation, microglial polarization, vascular protection, and reduction in oxidative stress. Across diverse models, it has been shown to decrease infarct size, preserve blood–brain barrier integrity, and enhance functional recovery. However, results also indicate variability depending on comorbid conditions and immune context, with some studies reporting limited or context-dependent benefits. Importantly, mechanistic insights suggest that fingolimod acts through both S1P receptor–mediated pathways and broader immunoregulatory effects, positioning it as a uniquely versatile agent. These findings strongly support translational exploration but also underscore the need to account for patient heterogeneity and comorbidities when moving from animal models to clinical application.

## 7. Clinical Evidence

Several clinical trials have been conducted to assess the therapeutic potential of fingolimod in acute ischemic stroke (AIS), with promising results.

In a 2014 pilot study by Fu et al., twenty-two patients with anterior circulation occlusion who presented beyond the 4.5 h window for thrombolytic therapy were randomized to receive either standard care alone or in combination with oral fingolimod (0.5 mg daily for three days). Patients treated with fingolimod demonstrated significantly better neurological outcomes during the acute phase, particularly within the first week, as reflected by greater reductions in NIHSS scores compared to controls. Additionally, lesion progression on imaging was less pronounced in the fingolimod group. Notably, individuals with total or partial anterior circulation occlusions appeared to derive more substantial benefit than those with lacunar infarcts. These improvements in neurological function and recovery remained evident at 90 days, as evaluated by the NIHSS, the modified Barthel Index (mBI), and the modified Rankin Scale (mRS), suggesting that fingolimod may have a lasting positive effect on post-stroke rehabilitation [42].

The subsequent study, published by Zhu et al. in 2015, explored the potential benefit of combining fingolimod with recombinant tissue plasminogen activator (rtPA) in patients eligible for thrombolytic therapy [14]. A total of 47 individuals were enrolled, with a subset receiving 0.5 mg of oral fingolimod daily for three days alongside rtPA. This combination resulted in notable clinical improvements: patients in the fingolimod group had significantly reduced lesion expansion during the first week, as well as lower NIHSS scores as early as the first day post-treatment. Furthermore, long-term assessments at 90 days indicated enhanced functional recovery. Importantly, fingolimod co-administration appeared to limit the extent of hemorrhagic transformation, reducing the size of cerebral bleeding observed in treated patients [14].

Building on earlier findings regarding the benefit of combining fingolimod with thrombolysis, Tian et al. (2018) conducted a prospective, multicenter, randomized, open-label study to explore its use in patients treated with alteplase beyond the conventional 4.5 h window [13]. A total of 46 patients, all presenting with anterior circulation occlusions and a perfusion mismatch confirmed on imaging, were assigned to receive either alteplase alone or in combination with oral fingolimod [13]. Notably, those in the combination group demonstrated significant neurological improvement within 24 h, as reflected by both NIHSS and mRS scores. Imaging data supported these clinical findings, revealing smaller perfusion lesions and reduced infarct expansion. Fingolimod also appeared to enhance reperfusion dynamics, improving both anterograde and retrograde flow. While the incidence of asymptomatic intracranial hemorrhage was higher in the combination group, no significant differences in symptomatic hemorrhage or infection rates were noted. Overall, this trial supports the idea that fingolimod may help extend the therapeutic window for thrombolytic therapy in AIS and promote better early and long-term neurological outcomes [13].

In a randomized clinical trial published in 2019, Zhang et al. investigated the effects of combining oral fingolimod (0.5 mg daily for three days) with standard thrombolytic therapy in acute ischemic stroke patients. Ninety individuals were enrolled, with half receiving the adjunct fingolimod treatment. While early assessments (day 14) revealed no significant differences between groups, by 90 days the fingolimod group showed better functional outcomes, with lower NIHSS and mRS scores and higher BI scores. MRI analysis also demonstrated reduced infarct volumes at both day 1 and day 7. Fingolimod led to transient lymphocyte reductions, consistent with its immunomodulatory effects. No cases of hemorrhagic transformation were reported in the combination group, and adverse events—including gastrointestinal and respiratory infections—did not differ significantly between groups. These findings support fingolimod’s potential to enhance long-term recovery when paired with alteplase, Table 2 [11].

Fingolimod as an Adjunct to Endovascular Treatment in Acute Ischemic Stroke: A Phase II Study.

This phase II, proof-of-concept clinical trial (NCT04629872) investigates the potential of Fingolimod, a sphingosine-1-phosphate receptor modulator, to enhance outcomes in patients undergoing endovascular treatment for acute anterior circulation ischemic stroke within 6–24 h of onset. The study includes adults aged 18–80 with confirmed large vessel occlusion and infarct core volumes between 15 and 70 mL.

Fingolimod is administered orally prior to thrombectomy, with the primary endpoint being the assessment of collateral circulation quality via a multiphasic contrast-enhanced CT scoring system. The intervention aims to modulate post-stroke neuroinflammation, potentially improving cerebral perfusion and outcomes.

Exclusion criteria include cardiac conduction disorders, infection, hepatic injury, or prior significant disability. If successful, the trial may support Fingolimod as a neuroprotective adjunct to mechanical reperfusion in ischemic stroke [43].

The FAMTAIS trial investigates the efficacy and safety of combining Fingolimod, an immunomodulatory agent, with standard bridging therapy (intravenous alteplase followed by mechanical thrombectomy) in patients with acute ischemic stroke (AIS) due to large vessel occlusion (LVO). Despite advances in reperfusion therapy, about 50% of patients remain functionally disabled, likely due to reperfusion injury and inflammation.

This randomized, multicenter, open-label study includes 98 participants, aiming to detect a ≥15% improvement in penumbra tissue salvage index with Fingolimod use. Primary and secondary endpoints assess infarct evolution, neurological recovery, and hemorrhagic complications. If successful, the trial could establish Fingolimod as an effective adjunct therapy to limit reperfusion-induced damage and enhance neurological outcomes post-stroke [44].

In sum, clinical studies suggest that fingolimod holds promise as an adjunctive therapy in acute ischemic stroke, particularly when combined with reperfusion strategies such as thrombolysis or thrombectomy. Early-phase trials consistently demonstrate improved neurological outcomes, reduced lesion growth, and lower rates of hemorrhagic transformation in patients receiving fingolimod in addition to standard care. These benefits appear most evident in large vessel occlusions and in settings where treatment is administered within the therapeutic window or slightly beyond it, thereby potentially extending eligibility for reperfusion therapies. Importantly, fingolimod has been generally well tolerated, with adverse events such as infection or cardiac disturbances remaining manageable. Ongoing larger-scale trials, including those testing its role alongside thrombectomy, will be critical to confirm efficacy, refine patient selection, and establish fingolimod’s place in the clinical management of stroke.

## 8. Side Effects and Safety of Fingolimod

Fingolimod has been an established therapy for multiple sclerosis (MS) since its Food and Drug Administration (FDA) approval in 2010. However it is interaction with the diverse and systemically expressed S1PRs can trigger broad signaling alterations. This can unfortunately trigger off-target effects due to its non-selective binding, particularly to S1PR3. Clinically, these side effects include an elevated risk of bradycardia, vascular complications, and bronchoconstriction, significantly limiting FTY720’s broader application [45]. Its therapeutic efficacy often surpasses the risks, even with these safety considerations, highlighting the crucial need to always assess the risk-benefit balance. The adverse effect profile and safety data of FTY720 have been thoroughly documented by Novartis, Switzerland. Common side effects reported with a 0.5 mg dose include various infections (like influenza, sinusitis, herpes zoster, and diarrhea), headaches, coughing, macular edema, and elevated liver enzymes (ALT and AST) of which the most serious concerns are cardiac conduction abnormalities, such as atrioventricular block [45].

In the context of ischemic stroke, clinical trials have utilized this drug alongside alteplase and several adverse events were reported. Common concerns included gastrointestinal bleeding and suspected pulmonary and urinary tract infections. Specific cardiac events, such as atrial fibrillation, were observed in a few instances, all of which resolved with standard medical intervention. Similarly, some cases of thrombocytopenia (low platelet count) occurred but resolved spontaneously. While suspected lung and urinary tract infections were noted, their rates were comparable between the fingolimod combination group and the alteplase-only group. Furthermore, though suspected lung infections were seen in a portion of fingolimod-treated patients, these were typically mild and responded promptly to antibiotic therapy.

Advanced Fingolimod delivery technologies hold significant promise in enhancing of its safety by precisely targeting therapeutic agent, thereby minimizing off-target exposure and consequently reducing the risk of adverse reactions.

In an effort to overcome biocompatibility limitations commonly associated with inorganic nanocarriers, recent work has introduced macrophage membrane-coated manganese dioxide (MnO_2_) nanoparticles as a novel delivery system for fingolimod. This biomimetic design not only prolongs the drug’s half-life but also reduces the hemolytic effects typically linked to MnO_2_ particles [46]. Leveraging the natural targeting ability of macrophage membranes, these nanocarriers selectively accumulate in ischemic brain regions by interacting with adhesion molecules upregulated on damaged endothelial cells [46]. Once internalized, the MnO_2_ core scavenges excess hydrogen peroxide, generating oxygen to alleviate local hypoxia and oxidative stress [46]. Simultaneously, the controlled release of fingolimod within acidic lysosomes supports microglial polarization from the pro-inflammatory M1 state to the reparative M2 phenotype [46]. Altogether, this multifunctional nanomedicine holds promise for addressing multiple injury mechanisms in ischemic stroke, including inflammation, oxidative damage, and impaired neuronal viability.

Recent advancements in drug delivery strategies have led to the development of a neutrophil membrane-coated polyprodrug nanosystem capable of selectively targeting ischemic brain regions. This biomimetic platform responds to heightened levels of reactive oxygen species (ROS) in the damaged tissue by releasing fingolimod (FTY720) directly at the site [47]. Notably, this approach enhances drug penetration across the blood–brain barrier and achieves over 15 times higher brain accumulation compared to free drug administration, while simultaneously minimizing systemic toxicity, particularly cardiovascular side effects [47]. Moreover, single-cell transcriptomic profiling revealed that the nanomedicine effectively suppresses post-ischemic neuroinflammation by shifting microglial activation toward a reparative phenotype. This immunomodulatory effect appears to involve downregulation of NLRP3 inflammasome activity and reduced C-X-C Motif Chemokine Ligand 2 (CXCL2) chemokine release, processes governed by the transcription factor Cebpb [47].

Building upon this innovation, another bioinspired nanoplatform has been engineered to further enhance targeted delivery in AIS. This system utilizes platelet membrane-coated nanobubbles assembled at a gas–liquid interface and loaded with fingolimod (FTY720). The resulting nanobubbles (PFNBs) display an organized spatial distribution of lipid and drug components, improving the presentation of targeting ligands and drug molecules on their surface [48]. This structure enables sequential targeting of ischemic lesions, more effective crossing of the blood–brain barrier, and selective internalization by activated microglia. Whole-genome transcriptomic analysis revealed that PFNBs contribute to both vascular stabilization and modulation of post-stroke inflammation, ultimately minimizing neural injury. These results highlight the therapeutic potential of modular nanocarriers in addressing multiple aspects of stroke pathology through synergistic targeting [48].

Importantly, although fingolimod is generally well tolerated and has an established safety record in multiple sclerosis, its non-selective binding to S1P receptors can cause off-target effects such as bradycardia, infections, and hepatic dysfunction, which remain relevant concerns in stroke populations. Clinical trials in ischemic stroke suggest that most adverse events are mild to moderate and manageable with standard care, though vigilance for cardiac and infectious complications is required. Importantly, recent advances in drug delivery—such as macrophage-, neutrophil-, and platelet-membrane nanocarriers—offer innovative ways to improve targeting, enhance brain penetration, and reduce systemic exposure. These strategies not only minimize side effects but also strengthen fingolimod’s therapeutic potential, reinforcing the importance of coupling efficacy with safety in the development of adjunctive treatments for stroke.

## 9. Results

The synthesized literature reveals a compelling body of evidence supporting the neuroprotective potential of fingolimod in ischemic stroke, derived from both experimental models and human clinical trials.

Preclinical Evidence: Multifaceted Mechanisms of Protection

Preclinical studies in various animal models of ischemic stroke (including transient and permanent middle cerebral artery occlusion—tMCAO/pMCAO, embolic MCAO—eMCAO, and photothrombosis—PT) consistently demonstrate that fingolimod administration leads to improved outcomes. The key findings across these studies include: Reduced Infarct Volume and Edema: Multiple studies reported a significant reduction in infarct size and cerebral edema following fingolimod treatment [26,32,36]. Improved Functional Recovery: Enhanced neurological scores (e.g., Longa score) and motor outcomes were frequently observed, with some studies also noting improvements in memory and synaptic plasticity [26,33,41]. Modulation of Neuroinflammation: A central mechanism of action is the potent anti-inflammatory effect. Fingolimod treatment was associated with:

Suppression of pro-inflammatory cytokines (IL-1β, IL-6, TNF-α) [26,31]. Inhibition of key inflammatory pathways, notably the HMGB1/TLR4/NF-κB axis [26,37]. Promotion of microglial polarization from the pro-inflammatory M1 phenotype towards the reparative M2 phenotype [33,35]. Blood–Brain Barrier (BBB) Preservation: Fingolimod was shown to protect BBB integrity by preventing the redistribution of tight junction proteins, thereby reducing vasogenic edema and the risk of hemorrhagic transformation (HT) [32,34].

Immunomodulation: The drug effectively reduces the infiltration of peripheral lymphocytes into the brain and enhances the frequency and suppressive function of regulatory T cells (Tregs), modulating the systemic immune response post-stroke [27,28,34]. Other Mechanisms: Additional protective mechanisms include the reduction in neuronal autophagy and apoptosis via the mTOR/p70S6K pathway [36] and the alleviation of oxidative stress [46]. The efficacy of fingolimod was found to be dose-dependent and could be influenced by the specific stroke model and the presence of comorbidities (e.g., diabetes, aging), with some studies showing attenuated effects or model-specific discrepancies [29,31].

### 9.1. Synergy with Reperfusion Therapies

A significant finding from preclinical research is the enhanced efficacy of fingolimod when used as an adjunct to thrombolysis. In an embolic MCAO model, the combination of fingolimod and alteplase significantly improved cerebral reperfusion and collateral flow, as visualized by advanced imaging techniques, surpassing the effects of alteplase alone [30].

### 9.2. Clinical Evidence: Translation to Human Studies

Clinical trials, though primarily small-scale and pilot in nature, corroborate the potential benefits of fingolimod in acute ischemic stroke (AIS), particularly in combination with standard reperfusion therapy. Improved Neurological and Functional Outcomes: Patients receiving fingolimod (typically 0.5 mg orally for 3 days) alongside alteplase demonstrated significantly greater improvements in NIHSS scores within the first week and better functional outcomes on the modified Rankin Scale (mRS) and Barthel Index (BI) at 90 days compared to controls receiving standard care alone [11,13,14,42].

Radiological Benefits: Combination therapy was associated with reduced lesion expansion on MRI, smaller final infarct volumes, and limited perfusion deficits [13,14]. Reduction in Hemorrhagic Complications: A key clinical benefit is the apparent ability of fingolimod to reduce the risk and extent of hemorrhagic transformation associated with thrombolysis, a finding consistent with its BBB-stabilizing effects observed preclinically [14]. Extension of Therapeutic Window: The combination of fingolimod with alteplase showed promise even when administered beyond the conventional 4.5 h window (up to 6 h), improving reperfusion dynamics and neurological recovery [13].

Safety Profile: In the context of stroke trials, fingolimod was generally well-tolerated. Adverse events, including transient lymphopenia, minor infections, and isolated cardiac events (e.g., atrial fibrillation), were manageable and their incidence was often comparable between treatment and control groups [11,13,14].

### 9.3. Emerging Delivery Systems

Recent research has focused on innovative nanotechnologies to enhance fingolimod’s targeted delivery and mitigate systemic side effects. Biomimetic delivery systems, such as: platelet membrane-coated fingolimod nanobubbles (PFNBs) for sequential targeting of ischemic lesions and the BBB [48]. Macrophage membrane-coated MnO_2_ nanoparticles that scavenge ROS and alleviate hypoxia while releasing fingolimod [46]. Neutrophil membrane-camouflaged polyprodrug nanoreactors that respond to the inflammatory microenvironment for site-specific drug release [47]. These advanced systems have demonstrated significantly higher brain accumulation of fingolimod (over 15-fold in some cases) and superior efficacy in modulating neuroinflammation and reducing infarct volume in preclinical models, while concurrently reducing systemic cardiovascular side effects.

In summary, the results from preclinical and clinical investigations converge to indicate that fingolimod exerts significant neuroprotection in ischemic stroke through multimodal mechanisms—primarily immunomodulation, anti-inflammation, and BBB stabilization. Its efficacy is enhanced when combined with reperfusion therapies like alteplase, improving both radiological and clinical outcomes while potentially mitigating associated risks.

## 10. Discussion

The collective evidence from preclinical and clinical studies positions fingolimod as a promising multi-targeted neuroprotective agent for ischemic stroke. Its primary mechanism of action—functional antagonism of S1P receptors—effectively deciphers the complex “thrombo-inflammatory” cascade that characterizes secondary brain injury. By sequestering lymphocytes within lymphoid organs, fingolimod directly attenuates the pivotal driver of delayed neuroinflammation: the infiltration of peripheral immune cells. This review demonstrates that this immunomodulation translates into tangible benefits, including reduced infarct volume, preserved BBB integrity, dampened neuroinflammation, and improved functional recovery in animal models.

The most compelling clinical finding is the synergistic effect observed when fingolimod is adjunct to reperfusion therapies, notably alteplase. The consistent reports of reduced hemorrhagic transformation across studies are of paramount importance, as this addresses a major limitation of thrombolysis [14,16]. Furthermore, the potential to extend the therapeutic window for alteplase administration, as suggested by Tian et al. (2018), could significantly increase the number of eligible patients and improve outcomes in delayed presentations [13].

However, the translation of these findings is not without challenges. The efficacy of fingolimod appears context-dependent, influenced by factors such as the stroke model (e.g., transient vs. permanent occlusion), the presence of comorbidities (e.g., diabetes), and the timing of administration [29,31]. The paradoxical finding that T-cell depletion worsened outcomes in a neonatal model underscores the delicate balance of the post-stroke immune response and the potential for off-target effects when modulating it systemically [38]. The drug’s non-selective S1PR binding profile, responsible for side effects like bradycardia, remains a clinical concern that may limit its widespread use [45].

This is where emerging biomimetic nanotechnologies offer a revolutionary path forward. The development of targeted delivery systems (e.g., PFNBs, macrophage-mimicking nanoparticles) represents a paradigm shift, aiming to maximize therapeutic efficacy in the brain while minimizing systemic exposure and associated adverse effects [46,47,48]. These innovations could potentially overcome the key translational barriers that have plagued previous neuroprotective agents.

In conclusion, while fingolimod presents a highly promising therapeutic strategy, its future in stroke care hinges on two critical avenues: first, the validation of its efficacy and safety in large-scale, multicenter randomized controlled trials like the ongoing FAMTAIS trial [44], and second, the continued advancement of targeted delivery platforms to harness its neuroprotective potential safely and effectively.

## 11. Limitations

### 11.1. Limitations of Major Clinical Trials

Despite encouraging early-phase findings, several limitations are evident across the major clinical trials evaluating fingolimod in acute ischemic stroke.

Most trials involved small sample sizes, limiting statistical power and generalizability. For example, the study by Fu et al. (2014) enrolled only 22 patients (11 in the treatment group), which restricts robust conclusions about efficacy or safety across diverse stroke populations [42]. Also the pilot trial by Zhu et al. (2015) compared fingolimod combined with alteplase to alteplase alone, but did not include a fingolimod-only arm, limiting the ability to assess the independent effect of fingolimod on clinical outcomes [14].

Another key aspect is that the heterogeneity in study protocols—including differences in dosing, time-to-treatment windows, stroke subtypes, and outcome measures—complicates cross-study comparisons.

In addition, many trials lacked long-term follow-up, making it unclear whether short-term benefits in perfusion or infarct volume translate to lasting functional improvements. In the Tian et al. (2018, Annals of Neurology) trial examining delayed alteplase plus fingolimod, imaging markers revealed improved reperfusion compared to alteplase alone, and while long-term outcomes were reported at 90 days via mRS, no data on extended functional recovery beyond that timeframe were provided [13].

Lastly, there is a potential for publication and selection bias, as most studies originate from a single research group or region, and predominantly report positive outcomes. This raises questions regarding reproducibility in larger, multicenter randomized controlled trials (RCTs) across varied clinical settings.

### 11.2. Limitations of This Review

As a narrative rather than systematic review, this article is subject to several methodological constraints. We did not follow formal protocols such as PRISMA, nor did we include a structured flowchart outlining the selection and screening of studies, which limits reproducibility. Study inclusion was based on the authors’ discretion rather than predefined criteria, introducing the potential for selection bias despite efforts to incorporate a diverse range of high-quality publications. Similarly, we did not apply standardized tools—such as ROBIS or the Newcastle–Ottawa Scale—to assess study quality or risk of bias, which may reduce the methodological rigor of our evaluation.

While we prioritized randomized controlled trials, meta-analyses, and expert consensus statements, lower-level evidence such as observational studies was not excluded when relevant, and some conclusions are necessarily drawn from these sources. We also acknowledge the risk of publication bias, given that studies with favorable results are more likely to be published and cited. However, to provide a balanced perspective, we intentionally included research with neutral or negative findings as well.

Finally, our review emphasizes literature from the past decade, offering a contemporary perspective but potentially overlooking older studies that may still hold value. Given the ongoing evolution of stroke research and immunomodulatory therapies like fingolimod, the conclusions presented here are based on evidence available up to June 2025. Readers are advised to consult updated sources to remain aligned with the latest developments and clinical recommendations.

## 12. Conclusions

Emerging evidence from both preclinical and clinical research supports the potential of fingolimod as a neuroprotective agent in ischemic stroke. Initially developed for multiple sclerosis, this S1P receptor modulator has demonstrated a range of beneficial effects in ischemic settings, including reduced infarct size, modulation of immune cell infiltration, and preservation of blood–brain barrier integrity. While the effectiveness of fingolimod in preclinical stroke models has varied, clinical trials have yielded promising results, with some indicating enhanced reperfusion and improved neurological recovery when fingolimod is used alongside thrombolytic therapy.

The intersection between immunomodulation and cerebrovascular protection underscores fingolimod’s relevance in stroke therapy, particularly in addressing the secondary injury mechanisms driven by inflammation. However, its role in routine clinical use remains to be clearly defined. Larger-scale randomized controlled trials are necessary to establish the safety, efficacy, and optimal treatment parameters of fingolimod in acute stroke care.

As the field of stroke research continues to evolve, integrating pharmacological advances with imaging and molecular diagnostics may further clarify fingolimod’s therapeutic window and mechanism of action. Continued interdisciplinary efforts will be crucial in determining whether fingolimod can transition from an experimental approach to a reliable adjunct in stroke management, ultimately contributing to improved patient outcomes.

## 13. Take Home Messages

✓ Neuroinflammation is a key target: Evidence from both preclinical and clinical studies consistently shows that post-stroke inflammation worsens neuronal injury, and fingolimod can modulate these pathways by reducing lymphocyte infiltration, preserving the blood–brain barrier, and promoting neuroprotection.
✓ Preclinical promise vs. clinical reality: Animal models show robust effects across diverse mechanisms (anti-inflammatory, vascular protection, microglial polarization), but translation to human patients remains partial, underscoring the need for more rigorous, multicenter trials.
✓ Adjunctive potential with reperfusion therapies: The strongest human data so far suggest that fingolimod, when combined with rt-PA or thrombectomy, improves functional outcomes and limits reperfusion injury, but its role as a monotherapy is less clear.
✓ Safety considerations remain critical: Fingolimod is generally well tolerated in stroke populations, though cardiac effects and infection risk warrant monitoring. Novel delivery systems may mitigate systemic adverse events.

Future research directions: Large-scale randomized trials, biomarker-driven patient selection, and nanotechnology-based formulations are essential to define fingolimod’s place in stroke management.

## Figures and Tables

**Table 1:** Overview of Post-2015 Preclinical studies on Fingolimod for Ischemic Brain Injury.

Title	Authors/Year	Model	Key Findings	Limitations
Fingolimod reduces inflammation via HMGB1/TLR4/NF-κB	Xing et al., 2024 [26]	tMCAO	↓ infarct, ↓ edema, ↓ cytokines, improved Longa scores	Rat model; short-term only; high doses
Fingolimod enhances Treg function	Malone et al., 2023 [28]	pMCAO	↑ Tregs, ↑ CCR8, ↑ CD4^+^ IL-10^+^, ↑ recovery	Only permanent model; single Treg timepoint
Fingolimod in aged and comorbid mice	Díaz Díaz et al., 2022 [29]	pMCAO	↓ infarct (ApoE^−^/^−^), ↑ motor (aged), ↑ T-cell modulation	No mechanistic analysis; no dose/timing range
Combined with alteplase improves reperfusion	Fu et al., 2014 [42]	eMCAO	↑ angiographic reperfusion and collateral flow	No behavioral/long-term data
Inflammation inhibited, but edema worsens	Li et al., 2020 [31]	tMCAO (diabetic)	↓ inflammation, ↑ edema, no functional benefit	Acute-phase only; BBB damage
Preserves tight junctions, ↓ inflammation	Wang et al., 2020 [32]	tMCAO	↓ microglia, ↓ apoptosis, ↓ infarct, preserved BBB	i.p. route only; indirect BBB leakage link
Enhances M2 microglia and angiogenesis	Shang et al., 2020 [33]	PT model	↑ M2 microglia, ↑ angiogenesis, ↑ recovery	Lacks apoptosis/BBB markers; PT model limits
Prevents hemorrhagic transformation	Salas-Perdomo et al., 2019 [34]	Reperfusion	↓ lymphocytes, ↓ HT (platelet-dependent)	No long-term or functional follow-up
M1→M2 via SphK2/HDAC1-KLF4 axis	Ji et al., 2019 [35]	In vitro/in vivo	↓ M1, ↑ M2 microglia, HDAC1 inhibition	24 h endpoint only; no in vivo behavior
T-cell depletion worsens neonatal HI injury	Herz et al., 2018 [38]	Neonatal HI	↓ CD4^+^/CD8^+^ T-cells, ↑ injury, ↑ innate cells	Single dose; neonatal-specific
S1PR3 mediates anti-inflammatory effects	Dong et al., 2018 [37]	OGD model	↓ HMGB1/TNF-α, ↓ TLR2/NF-κB, via S1PR3	Acute in vitro model only
↓ Autophagy via mTOR/p70S6K	Li et al., 2017 [36]	MCAO	↓ Beclin-1, ↑ mTOR, ↓ infarct, ↑ function	3-day follow-up; one regimen
Improves hippocampal plasticity & memory	Nazari et al., 2016 [41]	MCAO	↓ lesion, ↑ LTP/memory	Only memory tested; no dose/time variation
Maintains microvascular patency	Schuhmann et al., 2016 [40]	tMCAO	↓ infarct, ↑ function, maintained vessels	Short-term only; no synaptogenesis change
S1P exposure worsens stroke	Moon et al., 2015 [39]	MCAO	↓ infarct, ↓ inflammation, ↑ recovery	22 h only; one dose/timepoint

↓ decrease and ↑ increase.

**Table 2:** Fingolimod for Acute Ischemic Stroke: Clinical Trial Summary.

Trial	Research Type	Intervention	Patient No	Country	Primary Outcome	Safety/Adverse Events	Conclusion
Zhang (2019) [11]	RCTs	Fingolimod 0.5 mg orally, daily for 3 consecutive days + alteplase vs. alteplase + routine care	90	China	Significantly lower NIHSS scores, lower mRS scores, higher BI scores at 90 days (all with *p* < 0.05)	The major adverse events reported included gastrointestinal bleeding and suspected pulmonary and urinary tract infections.	Administration of fingolimod in combination with alteplase in patients with acute ischemic stroke was safe and led to significant improvements in NIHSS and mRS scores, as well as an increase in the BI score at 90 days post-treatment. The infarct volume was smaller in the group treated with the fingolimod + alteplase combination.
Tian (2018) [13]	RCTs	0.5 mg Fingolimod orally, daily for 3 consecutive days + alteplase vs. alteplase alone	46	China	Significantly greater improvement in NIHSS scores (*p* = 0.004), better mRS scores (*p* = 0.037) at 24 h post-treatment	Two incidences of atrial fibrillation, which resolved with the administration of amiodarone, and two incidences of thrombocytopenia, which resolved without intervention.	Fingolimod administered alongside alteplase beyond the standard 4.5 h window (4.5–6 h post-stroke onset) enhanced early neurological recovery, as evidenced by improved clinical scores. The combination reduced perfusion lesion size and inhibited infarct expansion (*p* < 0.001).
Zhu (2015) [14]	RCTs	Oral Fingolimod 0.5 mg daily for 3 consecutive days + alteplase vs. alteplase alone	47	China	Smaller lesion volume (10.1 vs. 34.3 mL, *p* = 0.04) Less hemorrhage (1.2 vs. 4.4 mL, *p* = 0.01) Improved NIHSS scores at day 1 (4 vs. 2, *p* = 0.02) Better functional recovery at day 90 (mRS 0–1: 73% vs. 32%, *p* < 0.01)	Suspected lung and urinary tract infections occurred at similar rates in both groups: 14% and 9% in the fingolimod plus alteplase group vs. 12% and 8% in the alteplase-only group.	Fingolimod combined with alteplase was well tolerated and led to reduced reperfusion injury, smaller lesion growth, and better short- and long-term outcomes in acute ischemic stroke patients treated **within 4.5 h of onset.**
Fu (2014) [42]	RCTs	Fingolimod 0.5 mg per day orally for 3 consecutive days + standard treatment vs. standard treatment or alone	22	China	Significantly milder neurological deficits at day 1 (median NIHSS 4 vs. 8 in control group) faster recovery by day 7 compared to the control group.	Suspected lung infections occurred in 27% of fingolimod-treated patients but were mild and resolved quickly with antibiotics.	Oral fingolimod administered **within 72 h of onset** in patients with acute anterior circulation ischemic stroke was safe and well tolerated. It significantly attenuated early neurological deficits, reduced infarct progression and microvascular permeability, and enhanced both short-term (first week) and long-term (90-day) functional recovery. These benefits were particularly pronounced in patients with anterior circulation occlusion.

## Abbreviations

NIHSS	National Institutes of Health Stroke Scale;
mRS	modified Rankin Scale;
BI	Barthel Index;
BBB	blood–brain barrier;
AIS	acute ischemic stroke;
LVO	large vessel occlusion;
rtPA	recombinant tissue plasminogen activator;
S1P	sphingosine-1-phosphate;
S1PR	sphingosine-1-phosphate receptor.
